# The Design, Development, and Clinical Assessment of a Novel Patented Laparoscopic Instrument for Ovariectomy in Dogs

**DOI:** 10.3390/vetsci12070639

**Published:** 2025-07-03

**Authors:** Marta Guadalupi, Claudia Piemontese, Caterina Vicenti, Rachele Piergentili, Francesco Staffieri, Luca Lacitignola

**Affiliations:** Sez. Cliniche Veterinarie e p.a., Dipartimento DiMePRe-J, Campus di Medicina Veterinaria, Università Degli Studi di Bari “Aldo Moro”, s.p. per Casamassima km 3, Valenzano, 70010 Bari, Italy; marta.guadalupi@uniba.it (M.G.); claudia.piemontese@uniba.it (C.P.); caterina.vicenti@uniba.it (C.V.); r.piergentili@studenti.uniba.it (R.P.); francesco.staffieri@uniba.it (F.S.)

**Keywords:** laparoscopy, ovariectomy, dog

## Abstract

Novel laparoscopic optical forceps (OFs), developed and patented by the University of Bari Aldo Moro (EP4119030, Bari, Italy), were designed to enhance safety and efficiency during laparoscopic ovariectomy in dogs by enabling atraumatic ovarian suspension and surgical maneuvers. This study aimed to describe the design, prototyping, sterilization validation, and preliminary clinical evaluation of this instrument. Prototypes were fabricated using ABS-like Pro resin via LSPc 3D printing. EtOx (ethylene oxide) sterilization proved to be the only effective method ensuring both microbiological safety and material integrity after 25 cycles. A randomized clinical trial involving 36 female dogs compared the OFs with conventional extracorporeal suture (ES) suspension using two-port laparoscopic ovariectomy. Surgical performance was assessed via operative times and complication rates. The OF group demonstrated significantly reduced ovariectomy (7.5 ± 1.6 min vs. 23.7 ± 7.6 min, *p* < 0.01) and overall surgical (14.2 ± 1.7 min vs. 30.4 ± 7.4 min, *p* < 0.01) times. No intraoperative complications occurred in the OF group, while the ES group exhibited instances of needle breakage, multiple suture attempts, and increased bleeding. The OFs enabled surgical maneuvers and ovarian suspension without requiring additional incisions or complex techniques, offering ergonomic advantages and compatibility with standard optics. These findings support the OFs as a promising tool to simplify and improve minimally invasive ovariectomy in veterinary surgery.

## Simple Summary

Laparoscopic surgery is widely used in veterinary medicine to reduce the invasiveness of procedures like ovary removal in dogs. However, existing techniques often require multiple incisions and expensive tools and may lead to complications such as bleeding or prolonged surgery times. This study aimed to develop and evaluate a new laparoscopic instrument that combines the function of a camera with a clamp to hold the ovary securely during surgery. The device was tested for reliability, sterilization methods, and clinical effectiveness. It was successfully produced using advanced 3D printing technology and was sterilized with ethylene oxide without damage, maintaining full functionality after repeated use. In a clinical trial involving 36 dogs, the new instrument significantly reduced surgery times compared to traditional techniques and minimized complications. It allowed the surgery to be performed with fewer incisions and without the technical difficulties associated with suturing. These results suggest that the new device can improve surgical safety, shorten operation times, and reduce the learning curve for surgeons. This development offers a more efficient and less invasive option for routine procedures, benefiting both veterinary professionals and animals.

## 1. Introduction

Over the past few decades, laparoscopic surgery has gained increasing acceptance in veterinary medicine, establishing itself as a valuable and less invasive alternative to conventional open laparotomy [1]. Among the most frequently performed minimally invasive procedures is ovariectomy (LOVE) in dogs and cats, which is primarily indicated for elective surgical sterilization [2].

Several laparoscopic variants of ovariectomy have been developed over time, including the one- [3], two- [1], and three-port techniques [4,5]; specialized single-port platforms that allow introduction of multiple instruments through a single incision [6,7,8,9,10]; transvaginal ovariectomy [11,12]; and the use of devices such as the T-LIFT [13]. The T-LIFT allows transabdominal organ retraction via a percutaneous introducer and a T-shaped anchor but requires abdominal wall penetration and may cause minor bleeding.

There is growing interest in reducing both the number and size of the portals used in minimally invasive procedures in order to minimize the surgical invasiveness [14]. However, reducing the number of cannulas can make a surgical procedure more difficult and can require a specific learning curve [8]. In addition, the severity of postoperative pain may be related to the number of trocars placed to access the abdominal cavity during the procedure, the nature of the surgery performed, and the experience of the surgeon [14]. Although these techniques are less invasive, they are not without technical difficulties and/or disadvantages and require the use of expensive equipment, such as specific telescopes with operating channels [3]. It should also be noted that in techniques involving ovarian suspension, this may be rather complicated in some patients. The needle used for suturing may damage the organ’s blood supply, causing bleeding that must be stopped and monitored and prolonging the surgery [15]. Furthermore, the needle could break or become damaged during its passage from the skin into the abdominal cavity and/or during its extraction, causing further problems. The passage of the needle could also be difficult in subjects with an excessively thick abdominal wall, for example due to a high BCS [3].

The aim of this study was to provide a technical description, develop, and perform a preliminary clinical evaluation of new laparoscopic optical forceps designed to facilitate atraumatic suspension and surgical maneuvers of the ovary during laparoscopic ovariectomy, improving the safety and efficiency of the procedure.

## 2. Materials and Methods

### 2.1. Instrumentation

**Figure 1:**
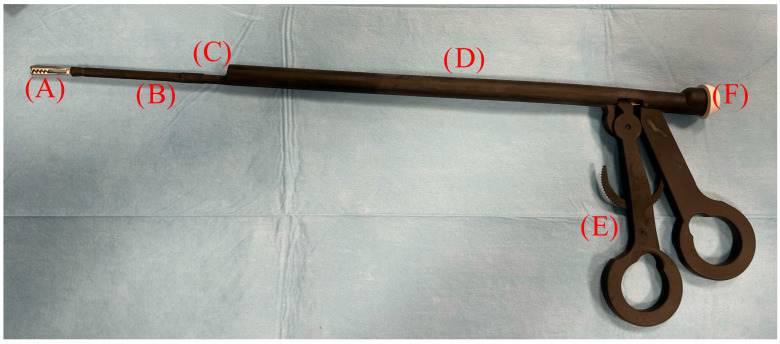
A highly reliable prototype of the optical forceps designed for minimally invasive laparoscopic procedures. The instrument was manufactured using 3D printing with Laser Projection Stereolithography (LPS) technology, employing an ABS-like biocompatible resin to ensure durability and structural precision. The main components are labeled as follows: (**A**) a grasping forceps tip designed for atraumatic tissue manipulation and ovarian suspension; (**B**) the shaft of the grasping forceps, which is housed within the main structure to allow controlled movement; (**C**) a 5.5 mm exit channel for insertion of a standard laparoscopic telescope, enabling direct visualization; (**D**) the main shaft, with an external diameter of 11 mm, serving as a supporting structure for both instruments; (**E**) an ergonomic handle designed to ensure intuitive control and stability during manipulation; (**F**) a protective plastic cap allowing safe and sterile insertion of the telescope into the optical channel.

The instrument under study is a laparoscopic device patented by the University of Bari (EP4119030; Bari, Italy) comprising a cylindrical tube with an outer diameter of 11 mm, into which a telescope can be inserted. At its distal end, there is an instrument in the form of a clamp, which can be operated by means of a handle located at the proximal end of the tube via a transmission element designed as a pull rod. The telescope insertion channel is equipped with a gasket, allowing the possibility of housing optics with a 5 mm diameter and a length equal to or greater than 29 cm. The device has a cylindrical central body with a diameter of 11 mm and a channel with a diameter of 5.5 mm into which the optics can be inserted. The manual control of the instrument is sized to fit the operator’s hand, as two of the operator’s fingers can be inserted into the handle to transmit an opening or closing movement to the gripping forceps by moving the fingers apart or closer together. The special configuration of the manual control and rack means that the instrument can be used by the operator with one hand, thus allowing the surgeon to use the other hand to maneuver a second instrument inserted through a second portal. The device is also equipped with a plastic cap with a diameter of 12 mm, which is made of a sealed material such as a silicone resin or a natural or synthetic rubber, preferably silicone resin, and positioned at the rear end. This cap forms the seal between the internal channel of the device into which the optics are inserted and the optics themselves, as it has a central hole and an outer edge through which it attaches to the body of the device. The gasket therefore prevents carbon dioxide from escaping from the inflated abdomen during the pneumoperitoneum procedure (Figure 1).

### 2.2. Production of Highly Reliable Prototypes

The first prototypes made it possible to identify the ideal materials, construction technologies, and dimensions for a final instrument to be tested in clinical trials. In particular, the prototypes were made from ABS-like Pro (acrylonitrile–butadiene–styrene) resin. The first prototypes made with ABS-like Pro resin were made using LSPc (Lubricant Sublayer Photo-curing) 3D printing technology based on technical drawings designed by the inventor (L.L.). LSPc is a high-speed, high-resolution vat photopolymerization method that employs an oxygen-permeable lubricating membrane to enable continuous printing without layer separation or suction forces. This process allowed for rapid fabrication of complex geometries with high dimensional accuracy and smooth surface finishes, which are critical for functional evaluation of laparoscopic instruments. The isotropic mechanical properties of parts produced with LSPc, combined with its compatibility with biocompatible and engineering-grade photopolymers, provided an optimal platform for producing functional prototypes suitable for preclinical validation.

### 2.3. Sterilization Tests

Sterilization tests were performed on the prototypes to test the life cycle, durability, and sterility of the material.

Sterilization cycles were performed as follows: method 1—autoclave sterilization at 134 °C for 10 min; method 2—low-temperature autoclave sterilization at 110° for 20 min; method 3—sterilization in 4% peracetic acid for 20 min; method 4—sterilization in ethylene oxide (EtOx) (Anprolene AN75, Andersen Sterilizers Inc., Haw River, NC, USA).

In addition, tests were performed to verify the presence of microorganisms on the instrument according to the following methods:

- ISO 18593:2004 for microbiological sampling of surfaces;
- Swabs with plastic shafts and cellulose tips with a transport medium and a disposable plastic delimiter (surface area: 100 cm^2^);
- Swabs taken from the handle, shaft, and insert of laparoscopic forceps after sanitization and EtOx sterilization;
- The total mesophilic count (TMC) at 30 °C (ISO 4833:2003—Horizontal method for the enumeration of microorganisms—Colony-count technique at 30 °C);
- The Enterobacteriaceae count (ISO 21528-2:2017, Part 2: Colony-count technique).

### 2.4. Ex Vivo Clinical Application

Experimental tests were carried out in a laparoscopic environment on cadavers and the clinical cases.

The tests on cadavers were performed on dogs that had died for reasons unrelated to this study, with the consent of the owners for the experimental tests to be carried out. Three intact female mixed-breed dogs that weighed ca. 15 kg with a BCS of 5/9 were included. The surgical procedure was performed as described in Section 2.5.2.

### 2.5. In Vivo Clinical Study

#### 2.5.1. Patients

This study was approved by the Ethics Committee for Veterinary Clinical and Zootechnical Studies of the Department of Precision and Regenerative Medicine and Jonian Area (Certificate of Approval No. 1729-2 III/13).

An initial pilot study was conducted on a limited number of clinical cases to obtain preliminary estimates of variability and effect size for the primary outcome measures. Based on these preliminary data, a formal power analysis was performed to determine the appropriate sample size required to detect clinically meaningful differences between the treatment groups.

The calculation indicated that a total of 36 animals (18 per group) would provide 80% power to detect statistically significant differences at a two-sided alpha level of 0.05. Following the pilot phase, the remaining cases were prospectively enrolled to reach the calculated sample size. This study was conducted on a cohort of 36 female dogs of various breeds with an average weight of 19.6 kg (±11.6, range: 4–44.3 kg) and a median BCS of 3/5 undergoing laparoscopic ovariectomy with the informed consent of their owners. The patients were assigned to two experimental groups according to a randomized list. One group involved the use of the two-port technique with ovarian suspension using external sutures (ES group), and one group involved the use of two ports and optical forceps (OF group). Therefore, 18 patients (*n* = 18) were assigned to each experimental group.

#### 2.5.2. Surgical Technique

All dogs were premedicated with intramuscular (IM) administration of 10 μg/kg of acepromazine (Prequillan; Fatro, Italy; 10 mg/mL) and, after 15 min, 0.3 mg/kg of methadone (Semfortan; Dechra, Italy; 10 mg/mL). When adequate sedation was achieved, a cephalic vein was cannulated for intravenous (IV) administration of fluids and drugs. General anesthesia was induced with propofol (Fresenius Kabi Propofol, 10 mg/mL) at 5 mL/kg IV, and the animals were intubated and connected to a re-breathing circuit. Anesthesia was maintained with inhaled isoflurane and pure oxygen (FiO_2_ 1). All dogs were mechanically ventilated during the entire procedure in volume-controlled mode (Servo-I; Maquet, Germany) with a tidal volume of 15 mL/kg, an inspiratory-to-expiratory ratio of 1:2, an inspiratory pause of 25% of the inspiratory time, and a PEEP of 5 cm of H_2_O. The respiratory rate (RR) was adjusted for the end-tidal carbon dioxide level (Pe’CO_2_), which was maintained between 40 and 55 mmHg.

All surgical procedures in both groups were performed by the same surgeon (L.L.), who has extensive experience in laparoscopic ovariectomy, including over 100 procedures per year using the extracorporeal suspension (ES) technique. Therefore, the complications observed in the ES group are unlikely to be attributable to operator inexperience and are more plausibly related to intrinsic limitations of the technique itself. The patients were placed in dorsal recumbency on a special reclining table. The abdominal skin was prepared aseptically, and the surgical field was delimited by sterile surgical drapes. The two-port technique was employed for both groups. A 12 mm port was then created 1 cm caudally to the umbilicus according to the modified Hasson technique. A 12 mm laparoscopic cannula was then inserted, a pneumoperitoneum was created at 8 mmHg, and a 5 mm laparoscopic camera with a 30° viewing angle (Hopkins II Optik 30°, Karl Storz, Tuttlingen, Germany) was inserted. An additional 5 mm port was created on the linea alba approximately 4–5 cm cranially to the umbilical port. Depending on the experimental group assigned in advance, in subjects belonging to the ES group, the ovary was suspended by extracorporeal suture with a 36 mm ½-circle needle and a USP1 nylon suture. Ovarian resection was performed using a radiofrequency coagulation device in all experimental groups. After resection of each ovary, it was extracted through the umbilical port. The contralateral ovary was dissected according to the same procedure, depending on the group to which it belonged.

**Figure 2:**
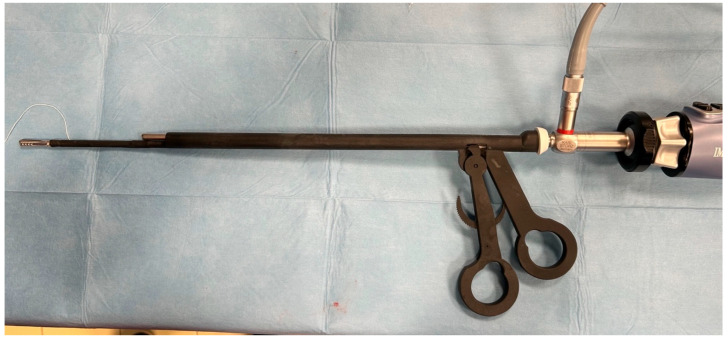
An image of the optical forceps prototype combined with the 5 mm telescope connected to the camera and light cable.

In the OF group, after placing the secondary portal, the OFs were inserted through the cannula with the telescope inside the insertion channel (Figure 2).

Then, the OFs were maneuvered for proper ovary manipulation and suspension, and the ovary was resected as described previously for the ES group. Ovary extraction was performed with the OFs by the central portal.

#### 2.5.3. Surgical Variables

The surgical times were recorded as follows: the cannula installation time (min), defined as the time from the first incision to the complete insertion of the second cannula; the ovariectomy time (min), defined as the time from the insertion of the second cannula to the extraction of the second ovary; and the closure time (min), defined as the time from the extraction of the second ovary to the placement of the final skin suture. The total surgery time (min) was calculated by summing the durations of all surgical phases. Any complications related to the surgical procedure were then evaluated, and bleeding from the ovary or pedicle was also assessed using a scale from absent to mild, moderate, or severe.

#### 2.5.4. Statistical Analysis

The data obtained were evaluated using JASAP statistical software (JASP Team 2024, JASP Version 0.19.3). The dataset was evaluated for a normal data distribution according to the Shapiro–Wilk test. Descriptive statistics were obtained from the data, and the surgical times in the two groups were compared using a one-way ANOVA test with alpha set at *p* < 0.05.

## 3. Results

### 3.1. Sterilization Test

Autoclave sterilization with moist steam according to method 1 (134 °C for 10 min) and method 2 (121 °C for 20 min) failed. In particular, all prototypes subjected to these methods underwent deformation that compromised their use in any form. Therefore, given the impossibility of performing functional tests, sterility tests were not carried out and methods 1 and 2 were excluded as sterilization methods for instruments made from this material.

Method 3, on the other hand, involved sterilization in peracetic acid. However, air bubbles were found inside the service channel of the optical forceps. This phenomenon was due to the technical characteristics of the instrument, and the creation of these air bubbles inevitably prevented the liquid from coming into contact with the internal surfaces of the instrument that house the laparoscopic optics. Although this sterilization technique did not alter the mechanical characteristics and there were no deformations that affected the functionality of the forceps, in terms of the repeatability of the sterilization performance, it is considered that this method has too many variables and does not guarantee safety in a clinical environment.

Method 4 involved sterilization with ethylene oxide. The ethylene oxide sterilization method involved preparing the instruments by first immersing them in an enzymatic solution for 15 min, followed by drying and placing the instrument in a sterilization bag. The sterilization protocol involved a 12 h ethylene oxide sterilization cycle at a temperature of 24 °C under vacuum.

This method was repeated on the instrument 25 times and proved to be highly reproducible, ensuring the same mechanical and functional performance of the instrument in each cycle without causing any defects or deformation of the instrument.

### 3.2. Microbiological Tests

The results of the microbiological analysis performed on the sampled surfaces demonstrated the effectiveness of the EtOx sterilization process, ensuring the complete elimination of the Total Microbial Load measured at 30 °C on the laparoscopic forceps.

The results obtained show that the sanitization phase carried out according to internal procedures is also effective in removing almost all microorganisms (Table 1).

The EtOx sterilization method is therefore considered the gold standard for ensuring long-lasting functionality exceeding 25 uses and microbiological safety during clinical use.

### 3.3. Cadaveric Tests

The cadaveric study made it possible to define the consistency of the highly reliable prototypes with regard to the dimensions, materials, and ergonomics developed during the project and to transfer them to a clinical laparoscopic setup that was completely comparable to the clinical surgical conditions experienced during clinical procedures in living patients. In fact, this phase was fundamental for testing the consistency of the instrument’s construction elements on tissues and organs by simulating laparoscopic ovariectomy surgeries and allowing for effective evaluation of the operating conditions. No particular or critical issues were identified during this phase, except for the need to rotate the instrument on its axis to prevent part of its shaft from partially obstructing the view of the laparoscopic optics. However, using laparoscopic optics with a 30° angle and rotating the handle of the forceps by a few degrees allowed the field of view of the surgical target to be widened and all surgical phases to be performed comfortably.

### 3.4. Clinical Study

In the clinical study, the tests performed in both groups allowed the procedure to be completed without the need to convert to open surgery. The OF instrument proved to be easy to handle and allowed easy integration with the optical system already in use. The pressure maintenance system proved sufficient to maintain the pneumoperitoneum pressure without gas leaks that could compromise the surgical maneuvers.

**Figure 3:**
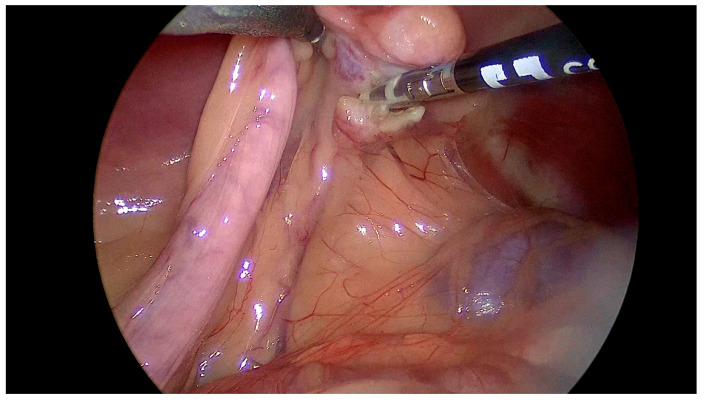
A representative intraoperative image illustrating the use of the optical forceps (OFs) during right ovary suspension and mesovarium transection utilizing a vessel-sealing device. The cranial direction is oriented to the right side of the image. The optical forceps, positioned on the left, are atraumatically suspending the right ovary, thereby enhancing the exposure of the mesovarium. Simultaneously, the vessel-sealing device, visible on the right, is employed to achieve hemostatic coagulation and precise transection of the mesovarian pedicle. This coordinated maneuver facilitates a safe and efficient dissection with optimal visualization of the vascular and ligamentous structures.

The ergonomics of the OF instrument during the manipulation of the ovarian pedicle and its resection using the vascular coagulation system allowed the maneuvers to be performed particularly easily without organ drop or bleeding during suspension. The removal of the ovary was also performed without difficulty or special maneuvers (Figure 3).

**Figure 4:**
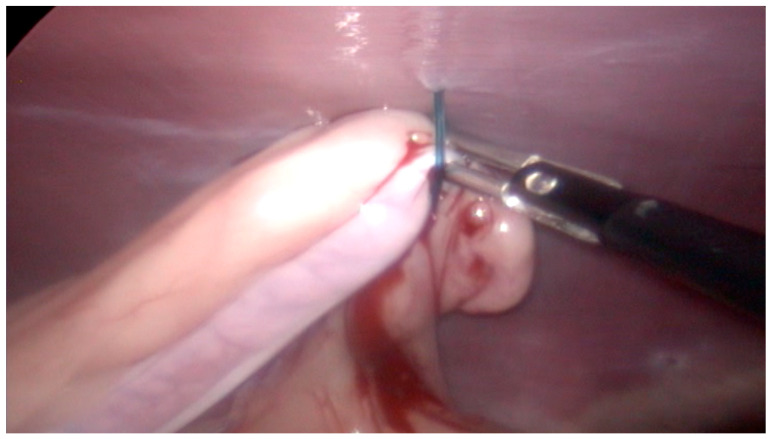
A representative intraoperative image from the extracorporeal suspension (ES) group during laparoscopic ovariectomy. The image, oriented with the cranial direction to the right, shows the ovarian suspension phase using a transabdominal suture. Mild but visible bleeding from the ovarian pedicle is evident at the needle passage site. This minor hemorrhage was promptly controlled and did not require additional intervention, but it illustrates one of the technical challenges associated with the ES technique.

However, in the ES group, we observed needle bending and breakage during the ovarian suspension phase in two cases of large-breed patients weighing more than 38 kg with a BCS of four. In addition, in some cases, several attempts were necessary to apply the suspension suture correctly, which prolonged the surgical time. Finally, in 50% of the subjects in the ES group, predominantly mild or moderate bleeding was observed, with only one case requiring electrocoagulation. Although well-controlled or minor, these complications required special attention from the surgeon, resulting in longer ovariectomy times (Figure 4).

**Figure 5:**
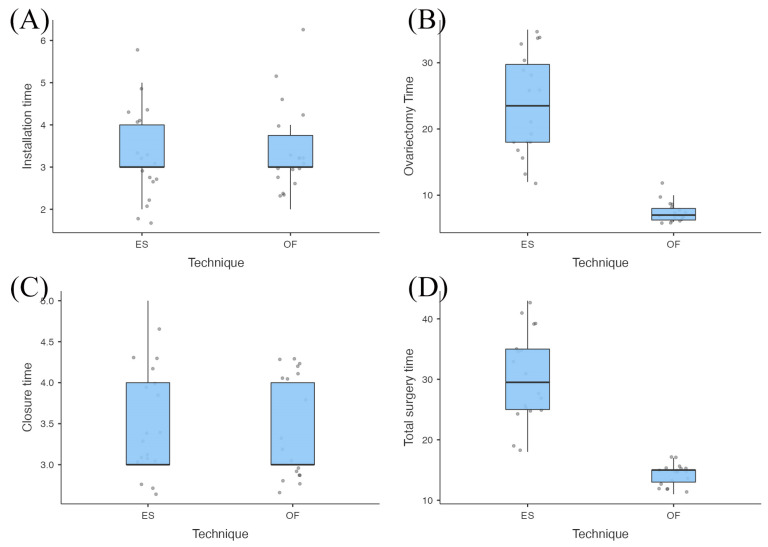
A box plot representation of the surgical phase durations comparing the extracorporeal suspension (ES) group and the optical forceps (OF) group. The panels illustrate the distribution of time (in minutes) for each surgical phase: (**A**) the installation time—the duration from initial port placement to completion of abdominal insufflation and instrument setup; (**B**) the ovariectomy time—the time required for bilateral ovarian resection, including vascular pedicle management; (**C**) the portal closure time—the time taken to close all laparoscopic access ports; (**D**) the total surgical time (skin to skin)—the cumulative time from the first skin incision to the final skin closure. Statistical analysis revealed significant reductions in both the ovariectomy time and the total surgical time in the OF group compared to the ES group (*p* < 0.01), suggesting enhanced procedural efficiency associated with the use of optical forceps for ovarian suspension.

With regard to the surgical times, although the installation and closure phases did not show statistically significant differences (*p* = 0.878 and *p* = 1.00, respectively), the ovariectomy times in the ES group were significantly longer than those in the OF group with a statistical significance of *p* = 0.001 (ES = 23.7 ± 7.6 min vs. OF = 7.5 ± 1.6 min). The significant reduction in the ovariectomy phase in the OF group significantly reduced the total surgery time (*p* = 0.001) (ES = 30.4 ± 7.4 min vs. OF = 14.2 ± 1.7 min). Table 2 and Figure 5 summarize the data obtained.

## 4. Discussion

This study evaluated the results of a preliminary study of a new patented laparoscopic instrument for use during ovariectomy in small animals.

This study consisted of several phases. In the first phase, the suitability of the materials used to create high-reliability models was evaluated.

The results show that an ABS-like Pro material is a versatile choice for the construction of the device. In fact, ABS-like Pro resin stands out for its remarkable impact resistance, making it ideal for applications that require durability. ABS-like Pro resin is characterized by its isotropic properties, ensuring that its mechanical qualities are uniform in all directions. This is essential for applications that require consistent performance regardless of the orientation of the part. Isotropy ensures greater reliability and predictability in the structural properties of components, making the resin particularly suitable for complex mechanical parts and engineering applications where material uniformity is essential. ABS-like Pro resin is valued for its ability to produce prints with smooth surfaces, reducing the need for post-processing. This is crucial for applications that require surface uniformity and prevention of biofilm formation. The smooth finish not only improves appearance but can also positively influence properties. This resin allows for a high level of detail in prints, making it perfect for complex and detailed projects. It is ideal for creating components with intricate geometries or models that require high fidelity to the original details. The improved formula ensures remarkable impact resistance, making prints more durable and suitable for intensive use. This makes it an excellent choice for parts that may be subjected to mechanical stress. ABS-like Pro resin has isotropic properties, meaning that its mechanical properties are uniform in all directions. This is crucial for applications that require uniformity and consistency in the mechanical properties of the material across all axes. ABS-like Pro resin is printed using the LSPc process, which is known for its efficiency and quality. In conclusion, it is an excellent choice for a high-quality resin with superior mechanical properties and has proven suitable for medical applications, while taking into account certain limitations such as low heat distortion and the visibility of support removal points. Its versatility and advanced features have made it an ideal solution for the construction of surgical instruments that require precision, strength, and durability. The possibility of creating prototypes using 316 L surgical steel was also evaluated. However, due to the technical difficulty of producing instruments with the characteristics required by the initial design; the production costs; and the sustainability of evolution, improvements, and reliability, this material was discarded from prototype production.

However, the choice of material limits the sterilization methods commonly used in clinical settings, reducing the sterilization options to methods that use low temperatures, such as ethylene oxide sterilization. However, this method is becoming increasingly popular in the veterinary field, and sterilization technologies are currently intended for end users or centralized sterilization services. The EtOx sterilization method ensured not only microbiological safety but also a tool life of more than 25 sterilization cycles. This allows the tool to be reused, significantly lowering the already low initial purchase costs.

Beyond the scope of the current application, the development of these novel laparoscopic forceps underscores the transformative potential of 3D printing in the field of minimally invasive surgery. Additive manufacturing enables rapid, place-based prototyping of highly specialized instruments that are tailored to the anatomical and procedural demands of a species and surgical indication. This approach not only facilitates the production of cost-effective and customizable tools but also decentralizes the manufacturing process, empowering surgical teams to innovate in-house and address specific unmet clinical needs. In the present study, this technology was employed to develop functional prototypes for clinical evaluation; however, the transition toward large-scale industrial production will be essential to ensure standardization, regulatory compliance, and broad availability of the device as an ‘off-the-shelf’ surgical instrument.

The endoscopic optical instrumentation used in the cadaver and clinical trials is commonly found in standard veterinary laparoscopic surgical equipment, thus avoiding the need for surgeons to purchase new specific instrumentation, as described previously [3]. In fact, the telescope used (5 mm diameter and 29 cm length) in the instrument is the most widely used in the field of small-animal laparoscopic surgery and is considered standard.

The device therefore reduces the number of incisions to be made in the animal during laparoscopic surgery to two while maintaining the maneuverability of the abdominal organs. In addition, it eliminates the need to suspend the ovary by suturing it to the abdominal wall, thus reducing the risks associated with this maneuver [15] and reducing the time required to perform it [16].

The use of a T-LIFT [13], on the other hand, avoids the use of a surgical half-circle needle, and the straight shape of the needle allows for easy insertion into the abdominal cavity, simplified maneuverability within the abdomen, and easy insertion into the ovarian ligament. It also makes it possible to choose the tension exerted on the ovary, which does not necessarily have to be kept close to the abdominal wall, as is essential during transcutaneous suturing. The results of this study demonstrate that the use of the optical forceps (OFs) during two-port laparoscopic ovariectomy (LapOVE) allows for a significant reduction in the operative time compared to techniques previously reported in the literature. In our study, the mean total surgical time was 14.2 ± 1.7 min, which was notably shorter than those reported in a recent prospective clinical trial evaluating the T’LIFT system and extracorporeal suspension (ES) sutures, where the mean operative times were 23.7 ± 9 min and 22.7 ± 5.5 min, respectively [13]. Importantly, while no complications related to ovarian suspension occurred in the OF group, the ES technique was associated with technical issues in large dogs, including needle breakage and failure to complete the suspension due to abdominal wall thickness. Although the T’LIFT device overcame these challenges, minor complications such as mesovary or skin bleeding were still observed. Finally, in dogs with a high BCS and an excessively thick abdominal wall, a suture needle may not pass effectively through the abdominal wall [3], a problem not encountered with the use of a T-LIFT [13].

In the three-portal technique, suspension of the ovary to the abdominal wall is not necessary, and surgical manipulation of the ovary is generally facilitated [1,2,14]. However, this approach requires a greater number of surgical incisions, which may contribute to increased postoperative pain and longer management times [5,14,17]. Compared to classical three-port techniques—whose reported operative times typically range from 22 to 40 min [4,18,19,20,21] depending on surgical expertise and the instrument configuration—the OF approach offers a notable ergonomic advantage by reducing the number of access ports without increasing technical complexity. The ability to simultaneously suspend the ovary and maintain visualization through the same device eliminates the need for transabdominal penetration or additional instruments, thereby minimizing risks and simplifying the procedure. Notably, the reduction to two portals is currently considered a standard technique for laparoscopic ovariectomy in dogs [1,4].

Thanks to the OF instrument, only two portals are necessary, and the surgeon can suspend the ovary in the abdominal cavity with the forceps and simultaneously use a second surgical instrument inserted into the second port, maintaining the view thanks to the optics inserted into the device. Moreover, the OFs allow surgical maneuvers for resection and extraction of a specimen with the same instrument.

A 30° laparoscope was selected over 0° optics in order to optimize the visualization of the surgical field, particularly given the proximity of the instrument’s shaft to the endoscopic channel. The angled view allowed the operator to dynamically adjust the visual axis and compensate for potential blind spots that may occur when using front-facing (0°) optics. This choice enhanced overall visualization without requiring repositioning of the instrument or the camera, thereby improving surgical ergonomics.

The results of the clinical study confirmed that the OF instrument allowed laparoscopic ovariectomy surgery to be performed in the recruited dogs in significantly less time than the standard two-port technique with ovarian suspension by extracorporeal suture performed in the ES group. Furthermore, when using the OF instrument, no complications related to ovarian suspension with a needle and a suture were observed, such as the need to make several attempts to apply the suture correctly for proper positioning, needle breakage or deformation, and bleeding from the ovary or ovarian pedicle during needle insertion, which were observed in the ES group. Although these complications were managed conservatively, they nevertheless demonstrated the need for the surgeon to extend the surgical time in order to monitor them and, in one case, even made electrocoagulation necessary to limit bleeding.

The channel that runs the length of the main body is unique in veterinary medicine in terms of its shape and the system connecting it to the body of the instrument itself, as it allows the insertion of any optics with 5 mm diameters, making it very versatile in the veterinary field.

This allows the surgeon to use instruments with which they are familiar, without the need to purchase new instruments, resulting in savings in terms of both time and money, as there is no need to learn how to use new surgical instruments.

This study has several limitations that should be acknowledged. First, all surgical procedures were performed by a single surgeon with extensive experience in laparoscopic ovariectomy, including the extracorporeal suspension (ES) technique. While this ensured standardization and minimized interoperator variability, it may limit the generalizability of the findings. The surgical performance of the optical forceps (OFs) in the hands of less experienced surgeons remains to be assessed. Future studies involving surgeons at various stages of the laparoscopic learning curve could provide valuable insight into the device’s usability, training potential, and impact on standardization across skill levels.

Second, mechanical strength testing, fatigue analysis, and material degradation assessment were not performed in this study, as the instrument evaluated was a high-reliability prototype produced using additive manufacturing. These aspects will need to be addressed once the final industrial-grade version of the instrument is developed. Dedicated mechanical validation and lifecycle testing will be essential to ensure long-term performance, safety, and compliance with regulatory requirements.

## 5. Conclusions

In conclusion, the novel instrument enables laparoscopic ovariectomy to be performed with only two abdominal incisions while preserving optimal maneuverability of the abdominal organs through the combined use of two operative instruments and a standard 5 mm laparoscope, as routinely employed in small-animal laparoscopy. A key advantage of this device lies in its ability to reduce the number of access ports required without necessitating significant modifications to the surgeon’s technique or the adoption of new procedural skills, thus facilitating its integration into current surgical practice.

This study provided a comparative analysis of the most widely adopted laparoscopic approaches in veterinary medicine, emphasizing their respective strengths and limitations and positioning the patented technique developed at the University of Bari Aldo Moro as a promising alternative. The instrument underwent thorough evaluation with respect to its construction materials and sterilization compatibility, ensuring both microbiological safety and structural integrity across more than 25 sterilization cycles. Preliminary in vivo results demonstrated the device’s safety, functionality, and ergonomic advantages, supporting its continued use in clinical settings. Further studies involving larger samples are planned to validate these findings and to explore the application of this device across a broader range of laparoscopic procedures.

## 6. Patents

“OPTICAL FORCEPS TO PERFORM LAPAROSCOPIC SURGERIES ON SMALL ANIMALS”, Università degli Studi di Bari “Aldo Moro” 70121 Bari (BA) (IT); Inventor: LACITIGNOLA, Luca I-70010 (IT); EP Number EP4119030A1.

## Figures and Tables

**Table 1:** TMC: total mesophilic count. Results in Colony Forming Units (CFU)/cm^2^. Samples were obtained post-sanitization with enzymatic solution and post-sterilization with 12 h ethylene oxide sterilization cycle.

Phase	Sample Point	TMC at 30 °C (CFU/cm^2^)	Enterobacteriaceae (CFU/cm^2^)
Post-sanitization	Handle	2	0
Post-sanitization	Shaft and insert	6	0
Post-sterilization	Handle	0	0
Post-sterilization	Shaft and insert	0	0

**Table 2:** Descriptive Analysis.

	Technique	Installation Time	Ovariectomy Time	Closure Time	Total Surgery Time
Mean	ES	3.28	23.7	3.44	30.4
	OF	3.33	7.50	3.44	14.3
Mean lower bound of 95% CI	ES	2.78	20.2	3.16	27.0
	OF	2.83	6.75	3.21	13.5
Mean upper bound of 95% CI	ES	3.77	27.3	3.73	33.9
	OF	3.83	8.25	3.68	15.1
Median	ES	3.00	23.5	3.00	29.5
	OF	3.00	7.00	3.00	15.0
Standard deviation	ES	1.07	7.69	0.616	7.43
	OF	1.08	1.62	0.511	1.74
Minimum	ES	2	12	3	18
	OF	2	6	3	11
Maximum	ES	6	35	5	43
	OF	6	12	4	17

## Data Availability

All data are included in the present manuscript.

## References

[1] Culp W.T., Mayhew P.D., Brown D.C. (2009). The effect of laparoscopic versus open ovariectomy on postsurgical activity in small dogs. Vet. Surg..

[2] Manassero M., Viateau V. (2018). Advances in laparoscopic spay techniques for dogs: The past, present and future. Vet. Rec..

[3] Dupre G., Fiorbianco V., Skalicky M., Gultiken N., Ay S.S., Findik M. (2009). Laparoscopic ovariectomy in dogs: Comparison between single portal and two-portal access. Vet. Surg..

[4] Van Goethem B.E., Rosenveldt K.W., Kirpensteijn J. (2003). Monopolar versus bipolar electrocoagulation in canine laparoscopic ovariectomy: A nonrandomized, prospective, clinical trial. Vet. Surg..

[5] Fuertes-Recuero M., de Segura I.A.G., López A.S., Suárez-Redondo M., Arrabé S.C., Hidalgo S.P., Fontanillas-Pérez J.C., Ortiz-Diez G. (2024). Postoperative pain in dogs undergoing either laparoscopic or open ovariectomy. Vet. J..

[6] Bydzovsky N.D., Bockstahler B., Dupré G. (2019). Single-port laparoscopic-assisted ovariohysterectomy with a modified glove-port technique in dogs. Vet. Surg..

[7] Gower S., Mayhew P. (2008). Canine laparoscopic and laparoscopic-assisted ovariohysterectomy and ovariectomy. Compend. Contin. Educ. Vet..

[8] Lacitignola L., Guadalupi M., Massari F. (2021). Single Incision Laparoscopic Surgery (SILS) in Small Animals: A Systematic Review and Meta-Analysis of Current Veterinary Literature. Vet. Sci..

[9] Tapia-Araya A.E., Díaz-Güemes Martin-Portugués I., Bermejo L.F., Sánchez-Margallo F.M. (2015). Laparoscopic ovariectomy in dogs: Comparison between laparoendoscopic single-site and three-portal access. J. Vet. Sci..

[10] Becher-Deichsel A., Aurich J.E., Schrammel N., Dupre G. (2016). A surgical glove port technique for laparoscopic-assisted ovariohysterectomy for pyometra in the bitch. Theriogenology.

[11] Arntz G.H.M. (2019). Transvaginal laparoscopic ovariectomy in 60 dogs: Description of the technique and comparison with 2-portal-access laparoscopic ovariectomy. Vet. Surg..

[12] Linhares M.T., Feranti J.P.S., Coradini G.P., Martins L.R., Martins A.R., Sarturi V.Z., Gavioli F.B., Machado Silva M.A., de Ataíde M.W., Teixeira L.G. (2019). Canine ovariectomy by hybrid or total natural orifice transluminal endoscopic surgery: Technical feasibility study and pain assessment. Vet. Surg..

[13] Delaune T., Matres-Lorenzo L., Bernarde A., Bernard F. (2021). Use of a T’LIFT transabdominal organ retraction device in two-portal laparoscopic ovariectomy in dogs. Vet. Surg..

[14] Case J.B., Marvel S.J., Boscan P., Monnet E.L. (2011). Surgical time and severity of postoperative pain in dogs undergoing laparoscopic ovariectomy with one, two, or three instrument cannulas. J. Am. Vet. Med. Assoc..

[15] Maurin M.P., Mullins R.A., Singh A., Mayhew P.D. (2020). A systematic review of complications related to laparoscopic and laparoscopic-assisted procedures in dogs. Vet. Surg..

[16] Manassero M., Leperlier D., Vallefuoco R., Viateau V. (2012). Laparoscopic ovariectomy in dogs using a single-port multiple-access device. Vet. Rec..

[17] Binder C., Katic N., Aurich J.E., Dupre G. (2018). Postoperative complications and owner assessment of single portal laparoscopic ovariectomy in dogs. Vet. Rec..

[18] Mayhew P.D., Brown D.C. (2007). Comparison of three techniques for ovarian pedicle hemostasis during laparoscopic-assisted ovariohysterectomy. Vet. Surg..

[19] Öhlund M., Höglund O., Olsson U., Lagerstedt A.-S. (2011). Laparoscopic ovariectomy in dogs: A comparison of the LigaSure™ and the SonoSurg™ systems. J. Small Anim. Pract..

[20] Van Nimwegen S.A., Kirpensteijn J. (2007). Comparison of Nd:YAG surgical laser and Remorgida bipolar electrosurgery forceps for canine laparoscopic ovariectomy. Vet. Surg..

[21] Van Nimwegen S.A., Van Swol C.F., Kirpensteijn J. (2005). Neodymium:yttrium aluminum garnet surgical laser versus bipolar electrocoagulation for laparoscopic ovariectomy in dogs. Vet. Surg..

